# External Validation of an MRI-Derived Radiomics Model to Predict Biochemical Recurrence after Surgery for High-Risk Prostate Cancer

**DOI:** 10.3390/cancers12040814

**Published:** 2020-03-28

**Authors:** Vincent Bourbonne, Georges Fournier, Martin Vallières, François Lucia, Laurent Doucet, Valentin Tissot, Gilles Cuvelier, Stephane Hue, Henri Le Penn Du, Luc Perdriel, Nicolas Bertrand, Frederic Staroz, Dimitris Visvikis, Olivier Pradier, Mathieu Hatt, Ulrike Schick

**Affiliations:** 1 Department of Radiation Oncology, CHRU Brest, 29200 Brest, France; francois.lucia@chu-brest.fr (F.L.); olivier.pradier@chu-brest.fr (O.P.); ulrike.schick@chu-brest.fr (U.S.); 2 LaTIM, INSERM, UMR 1101, CHRU Brest, 29200 Brest, France; martin.vallieres@mail.mcgill.ca (M.V.); visvikis@univ-brest.fr (D.V.); hatt@univ-brest.fr (M.H.); 3 Urology Department, CHRU Brest, 29200 Brest, France; georges.forunier@chu-brest.fr; 4 Medical Physics Unit, McGill University, Montreal, QC H3A 0G4, Canada; 5 Anatomopathology Department, CHRU Brest, 29200 Brest, France; laurent.doucet@chu-brest.fr; 6 Radiology Department, CHRU Brest, 29200 Brest, France; valentin.tissot@chu-brest.fr; 7 Urology Department, Cornouaille Hospital, 29000 Quimper, France; g.cuvelier@ch-cornouailles.fr; 8 Radiology Department, Cornouaille Hospital, 29000 Quimper, France; s.hue@ch-cornouailles.fr; 9 Radiology Department, Keraudren Clinique, 29000 Brest, France; contact@keraudren-grandlarge.fr; 10 Radiology Department, Clinique St Michel, 29000 Quimper, France; contact@polyclinique-quimpersud.fr; 11 Urology Department, Clinique St Michel, 29000 Quimper, France; cabinetducaphorn@orange.fr; 12 Anatomopathology Department, Ouest Pathologie, 29000 Quimper, France; info@anapath-quimper.fr

**Keywords:** magnetic resonance imaging, prostatic neoplasms, radiomics, machine learning, treatment failure

## Abstract

Adjuvant radiotherapy after prostatectomy was recently challenged by early salvage radiotherapy, which highlighted the need for biomarkers to improve risk stratification. Therefore, we developed an MRI ADC map-derived radiomics model to predict biochemical recurrence (BCR) and BCR-free survival (bRFS) after surgery. Our goal in this work was to externally validate this radiomics-based prediction model. Experimental Design: A total of 195 patients with a high recurrence risk of prostate cancer (pT3-4 and/or R1 and/or Gleason’s score > 7) were retrospectively included in two institutions. Patients with postoperative PSA (Prostate Specific Antigen) > 0.04 ng/mL or lymph node involvement were excluded. Radiomics features were extracted from T2 and ADC delineated tumors. A total of 107 patients from Institution 1 were used to retrain the previously published model. The retrained model was then applied to 88 patients from Institution 2 for external validation. BCR predictions were evaluated using AUC (Area Under the Curve), accuracy, and bRFS using Kaplan–Meier curves. Results: With a median follow-up of 46.3 months, 52/195 patients experienced BCR. In the retraining cohort, the clinical prediction model (combining the number of risk factors and postoperative PSA) demonstrated moderate predictive power (accuracy of 63%). The radiomics model (ADC-based SZE_GLSZM)_ predicted BCR with an accuracy of 78% and allowed for significant stratification of patients for bRFS (*p* < 0.0001). In Institution 2, this radiomics model remained predictive of BCR (accuracy of 0.76%) contrary to the clinical model (accuracy of 0.56%). Conclusions: The recently developed MRI ADC map-based radiomics model was validated in terms of its predictive accuracy of BCR and bRFS after prostatectomy in an external cohort.

## 1. Introduction

Prostate cancer (PCa) is the most common cancer among men with approximately 191,900 patients expected to be diagnosed in 2020 in the United States, and more than 33,000 deaths annually [1]. With excellent long-term outcomes, radical prostatectomy (RP) is a first-line treatment for localized PCa. [2]. Nevertheless, biochemical recurrence (BCR) occurs in 50% of these patients, especially those with high-risk features, such as locally advanced disease (T3-4), positive margins (R1), and high Gleason scores, with BCR being a surrogate of metastatic relapse and cancer-specific death [3]. Adjuvant radiotherapy (aRT) improves BCR-free survival, but its impact on overall survival is controversial and was shown to increase gastrointestinal and genitourinary side effects [4,5,6,7]. Hence, aRT was recently challenged by early salvage radiotherapy (esRT) with no significant benefit emerging for the use of aRT for BCR-free survival, thus highlighting the need for additional biomarkers to enable patient selection [8,9,10].

The natural history of relapse after RP is heterogeneous even in patients with high-risk features and may reflect a broad range of underlying tumor pathophysiological processes. Magnetic resonance imaging (MRI) is routinely used by clinicians for diagnosis and staging of PCa. In an attempt to move toward more quantitative exploitation of medical images, interest in the radiomics approach has recently been growing. Radiomics features are statistical, geometrical, or textural metrics providing quantitative measurements of tumor intensity, shape, and heterogeneity, which may reflect intratumoral histopathological properties and provide prognostic information in several pathologies, including PCa [11,12,13].

We previously reported on the development of a predictive model based on a single radiomics feature, size-zone emphasis (SZE_GLSZM_), extracted from ADC maps obtained from 107 pre-therapeutic prostatic diffusion weighted imaging (DWI) MRIs [14]. Grey-Level Size Zone Matrix (GLSZM) evaluates the size of zones of the same grey-level voxels. This model was trained on a cohort treated at our center. Through this study, we aimed to validate this model in an independent external cohort.

## 2. Methods

### 2.1. Selection of Patients

Patients who were histologically proven to be PCa patients and treated with RP with or without lymphadenectomy from 2010 to 2016 at one of two institutions, i.e., Institution 1: University Hospital of Brest and Institution 2: the Hospital of Quimper and the Clinique-St Michel in Quimper, were retrospectively considered. We only included patients harboring high-risk features on the pathologic specimen, namely, pT3a-b or pT4 and/or R1 and/or Gleason 8–10, and with available preoperative pelvic MRI.

**Figure 1:**
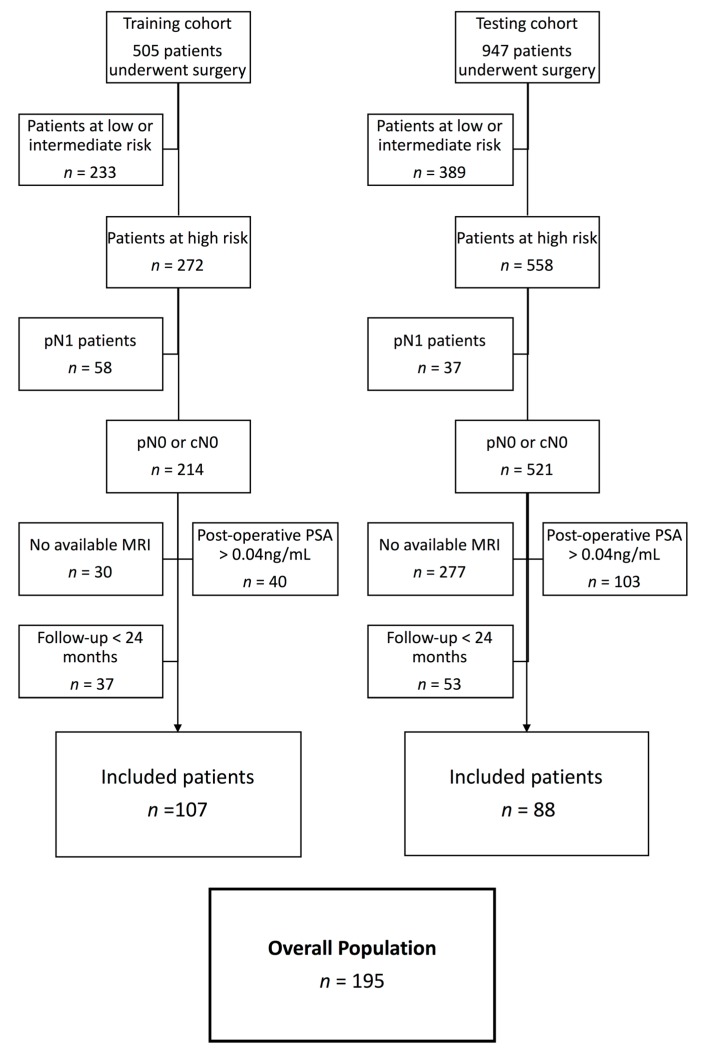
Flowchart of the selection. Abbreviations: pN1: lymph node involvement after lymphadenectomy; pN0: absence of lymph node involvement after lymphadenectomy; cN0: absence of lymph node involvement after clinical/radiological exam; PSA: Prostate Specific Antigen; MRI: Magnetic Resonance Imaging.

All patients with lymph node involvement after extensive lymphadenectomy were excluded, as were those whose PCa diagnoses were obtained after cystoprostatectomy for bladder carcinoma. We excluded patients who received adjuvant treatment (aRT and/or adjuvant androgen deprivation therapy) and those with postoperative detectable PSA (PSA > 0.04 ng/mL at 3 months following RP). Patients with unavailable MRI were also excluded. Patient selection is detailed in Figure 1.

Clinical and biological follow-ups were performed at 1, 3, and 6 months and then every 6 months after surgery. A minimum follow-up of 24 months was also mandatory, except in cases of BCR.

The patients from Institution 1 were already known to us. As mentioned above, our previous article dealt with the development of the predictive models [14]. For the current analysis, we carried out retraining of the predictive models using updated follow-up information and validation using the Institution 2 cohort.

This study was approved by both hospital ethical committees (PREBOP 29DRC18.0108) and all patients gave their consent for the use of their clinical and imaging data.

### 2.2. Endpoints

The primary endpoint was the prediction of BCR, which was defined as a PSA increase above 0.2 ng/mL confirmed by 2 successive blood samples. The secondary endpoint was the prediction of BCR-free survival (bRFS).

### 2.3. Surgery

Radical prostatectomy was performed by only one highly-experienced surgeon in Institution 1 and by 5 surgeons with diverse surgical experiences in Institution 2, all following EAU (European Association of Urology) guidelines [15].

Indication of lymphadenectomy was based on preoperative lymph node invasion risk assessment. Until 2015, extended lymphadenectomy was at the surgeon’s discretion. After 2015, extended lymphadenectomy was the standard of care.

### 2.4. MRI

Preoperative imaging differed between institutions. Four different MRI scanners were used, including a Phillips 3T (Philips Healthcare, Eindhoven, The Netherlands) and a Siemens 1.5T (Siemens Healthcare, Malvern, PA, USA) in Institution 1 and a Philips 1.5T (Philips Healthcare, Eindhoven, The Netherlands) and a Siemens 1.5T (Siemens Healthcare, Malvern, PA, USA) in the two clinical centers of Institution 2.

Acquisition was performed in supine position, using a 6-channel phased-array surface coil, following the European Society of Urogenital Radiology (ESUR) guidelines. Anatomical images (axial turbo spin echo T2-weighted) were combined with functional sequences, such as axial diffusion sequences using several *b*-values up to 1100 (Table S1) and dynamic contrast-enhanced sequences (perfusion sequence for Philips 3T and a T1 sequence with gadolinium injection for Siemens 1.5T and Philips 1.5T). ADC maps were calculated using each corresponding manufacturer’s software. Full details about acquisition parameters are provided in Table S1.

### 2.5. Clinical Features

We included the following clinical variables: T stage (extra-capsular extension and/or seminal vesicle invasion), Gleason score (surgical pathology only), pre- and postoperative PSA, margin status, age at surgery, number of risk factors, and CAPRA-S Score (Cancer of the Prostate Risk Assessment Score) [16]. For patients who did not undergo pelvis lymphadenectomy, the risk of lymph node involvement was evaluated using the Roach formula [17], i.e., risk of lymph node involvement (%) = PSA × 2/3 + (Gleason score – 6) × 10, with a risk of >15% being considered high.

### 2.6. Tumor Delineation

**Figure 2:**
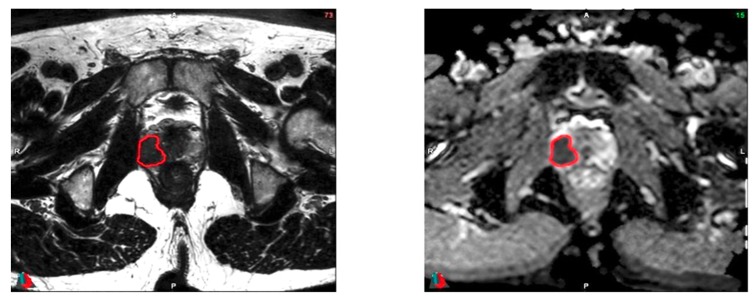
Examples of delineation on both T2 (left) and ADC (right) sequences. Images acquired on a Philips 3T Scan. Patient #27: Initial PSA (Prostate Specific Antigen) of 6.80 ng/mL, no clinical anomaly. MRI: suspicion of extracapsular extension. Pathology exam: Gleason score of 9 (4 + 5), bilateral invasion of the prostate (pT2c). Magnification scale: ×1.

Using the Fast GrowCut Effect extension available in 3D Slicer^®^ v4.8.0 index, prostatic tumors were semi-automatically delineated by a single expert (V.B.) on both the ADC and T2 sequences using all sequences available on the preoperative multiparametric MRI (see Figure 2).

### 2.7. Radiomics Features

Prior to the extraction of radiomics features, wavelet filters were applied to each MRI sequence, thereby creating 8 filtered images with high-pass and low-pass versions of the wavelet basis function coiflet1. Radiomics features were extracted using homemade radiomics code implemented in MatLab^®^, following the most up-to-date guidelines and benchmark values of the Image Biomarker Standardisation Initiative (IBSI) [18]. Only the previously identified feature (ADC SZE_GLSZM_) [14] was considered in the present study, as explained in the statistical analysis section below.

### 2.8. Statistical Analysis

In our previous work, we trained the model using 107 patients from Institution 1. First, we used a feature selection method based on stability, robustness, and intercorrelation checks [19] in order to only evaluate a reduced subset of features in the model training and validation. After training using two-thirds of the cohort and validating using the rest, and additionally combining the radiomics (kept after feature set reduction) and clinical variables, three predictive models were built, i.e., a radiomics model based on a single textural feature (ADC SZE_GLSZM_), a clinical model based on preoperative PSA and age at surgery, and a combined radiomics and clinical model [14].

In the present study, we chose to retrain the three models (clinical, radiomics, and clinical + radiomics) using the entire cohort of Institution 1 with updated follow-up data and to evaluate them in comparison to the external validation cohort of Institution 2.

The radiomics feature ADC SZE_GLSZM_ and all collected clinical variables (see clinical features) were then evaluated for their predictive ability with univariate (Receiver Operating Characteristic (ROC) curves) and multivariate (Cox regression) analyses in Cohort 1 (*n* = 107). Optimal cut-off values for each feature/model were defined using the Youden Index and models were built using a logistic combination of variables of interest. Models with their specific features and associated cut-off values were then evaluated using the testing cohort (*n* = 88). Quantitative performance evaluation was carried out using balanced accuracy (BAcc), sensitivity (Se), and specificity (Sp) regarding prediction of BCR via Kaplan–Meier curves and the log-rank test regarding bRFS stratification. All statistical analyses were performed using MedCalc v14.8.1.

We evaluated our study based on the radiomics quality score developed by Lambin et al. [20].

### 2.9. Harmonization Method

To pool radiomics features extracted from the four MRI scanners relying on different protocols, we used the harmonization statistical method ComBat [21], which was previously exploited to harmonize MRI data [22,23]. ComBat proved successful in removing intersite technical variability while preserving intersite biological variability.

As we did not apply the ComBat method in our previous work, a new cut-off value for ADC SZE_GLSZM_ was determined using the entire training cohort of Institution 1 after harmonization (since two different scanners were used). This resulted in two additional “harmonized” prediction models (radiomics only and combined clinical + radiomics).

### 2.10. Inter-Reader Variability

Two other experts (U.S. and F.L.) performed manual segmentation while blinded to the results of the previous delineation by V.B. in a subset (*n* = 15) of the testing cohort. The variations between each delineation were evaluated using the average Hausdorff distance and the DICE coefficient [24]. The variations of the radiomics feature (ADC SZEGLSZM) were quantified and intraclass correlation coefficients (ICC) were used to evaluate their agreement across different delineations. Additionally, the resulting impact on the actual classification of the patients was also reported.

## 3. Results

### 3.1. Patient Characteristics

In the retraining cohort of Institution 1, 505 patients underwent RP between January 2010 and December 2016. According to pathological analysis, 272 patients (54%) were categorized as high-risk of BCR (T3a/T3b or T4 and/or R1 and/or Gleason 8–10).

Overall, 107 patients were excluded because of positive lymph nodes (*n* = 58), follow-up < 24 months (*n* = 37), or postoperative detectable PSA > 0.04 ng/mL (*n* = 40). Among the remaining patients (*n* = 137), preoperative MRI was available in 107 (78%) (Figure 1).

In the external testing cohort of Institution 2, during the same period, 947 patients underwent RP, among whom 558 presented high-risk features. Eighty-eight patients were included; others were excluded for the following reasons: pN1 status (*n* = 37), postoperative PSA > 0.04 ng/mL (*n* = 103), follow-up < 24 months (*n* = 53), or unavailable MRI (*n* = 277).

Training and testing cohorts were comparable except for median bRFS, median follow-up, and surgical margin status, which all significantly differed (Table 1).

Overall, the majority of patients had pT3–pT4 disease (62%) and microscopically involved margins (68%). Seventeen percent of MRIs (*n* = 33) were acquired on a 3T scanner and 83% (*n* = 162) on a 1.5T scanner.

When comparing the analyzed population with the eligible population with no MRI (Table S2), substantial differences existed, especially regarding surgical margin status and bRFS in Institution 2, and follow-up in both institutions.

### 3.2. Outcome

In the training set (Institution 1), the median follow-up was 57.0 months (range: 24–107.4). Among the 107 patients, BCR occurred in 18 patients (17%) after a median bRFS of 49.2 months (7.3–107.4 months). Within the relapsing population and at the last follow-up, 11 patients (61%) experienced BCR alone and the remainder developed lymph node (3–17%) or distant metastases (4–22%).

In the testing set (Institution 2), after a median follow-up of 41.9 months (range: 24–102.4), 34 experienced BCR (39%), with a median bRFS of 33.0 months (range: 4–102.4).

In the subset of patients that did not undergo lymphadenectomy, lymph node involvement risk via the Roach Formula was evaluated to a median of 5% (CI95% of 4.4–6.4%).

### 3.3. Model Retraining

**Figure 3:**
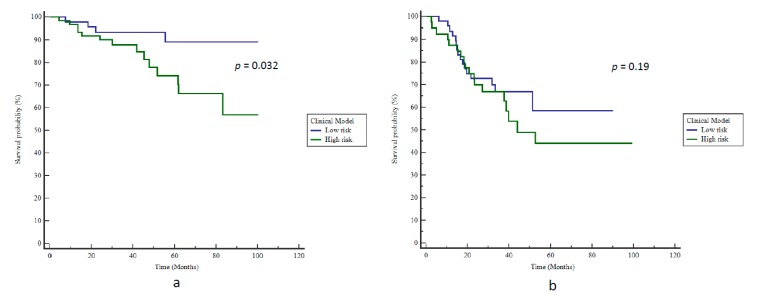
Kaplan–Meier estimates of biochemical relapse-free survival using the clinical model for (**a**) training and (**b**) testing cohorts.

In the univariate analysis, three clinical features were significantly correlated with BCR: A Capra-S Score > 3, the number of risk factors, and a postoperative PSA > 0.01 ng/mL. In the multivariate analysis, only ADC SZE_GLSZM_ remained independent and significantly correlated with BCR (Table 2). When focusing on clinical variables, the best result to predict BCR was obtained logistically by combining the number of risk factors and the postoperative PSA, thereby resulting in an AUC of 0.68 (*p* = 0.007) and a BAcc of 63% (Se 78%, Sp 47%) after applying the optimal cut-off value. Patients were stratified according to bRFS with an HR of 3.2 (*p* = 0.032, Figure 3a).

**Figure 4:**
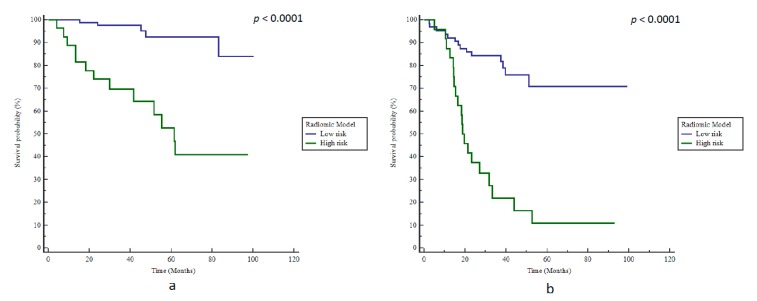
Kaplan–Meier estimates of biochemical relapse-free survival using the radiomics model in (**a**) training and (**b**) testing cohorts.

ADC SZE_GLSZM_ (AUC 0.82) with a cut-off value of 0.53 (the cut-off value determined in the initial development study was 0.528) [14] was the best model, with a BAcc of 79% regarding BCR predictions (Se 72%, Sp 85%, *p* < 0.0001) and a corresponding high stratification power according to bRFS with an HR of 8.7 (*p* < 0.0001, Figure 4a).

**Figure 5:**
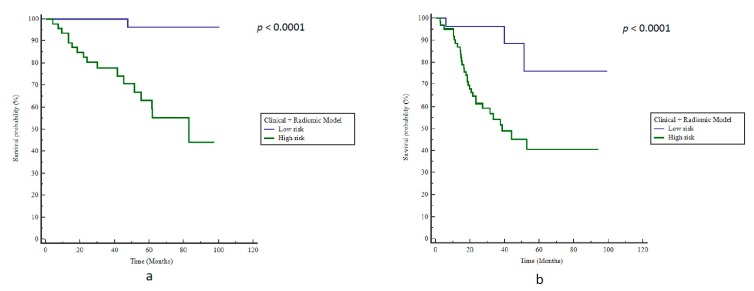
Kaplan–Meier estimates of biochemical relapse-free survival using the radiomics + clinical model in (**a**) training and (**b**) testing cohorts.

The model consisting of the logistical combination of clinical and radiomics features resulted in a BAcc of 84% (Se 94%, Sp = 67%, *p* < 0.0001) and a stratification for bRFS with a HR of 25 (*p <* 0.0001, Figure 5a).

The ROC curves corresponding to each training model can be found in Figure S1.

### 3.4. Model Evaluation in the Testing Cohort

The clinical model failed to validate the external cohort of Institution 2, with a BAcc of only 56% (Se 53%, Sp 59%) for BCR prediction; therefore, it was unable to stratify patients according to bRFS (*p =* 0.19, Figure 3b).

However, the radiomics model remained accurate with a BAcc of 76% (Se 59%, Sp 93%), and was able to stratify patients according to bRFS with an HR of 5.5 (*p <* 0.0001, Figure 4b). Patients with ADC SZE_GLSZM_ values below 0.53 had a median bRFS of 19.2 months compared to 37.0 months in patients above this cut-off (*p =* 0.0013). Furthermore, at the last follow-up, 83.3% of patients with ADC SZE_GLSZM_ values below 0.53 exhibited BCR compared to the 22% of patients with ADC SZE_GLSZM_ values above 0.53.

The combined radiomics + clinical model did not outperform the radiomics-only model, with an accuracy of 67% (Se 91%, Sp 43%) and a significant prediction of bRFS (HR 5.7, *p <* 0.0001, Figure 5b). The positive predictive value (PPV) and negative predictive value (NPV) were respectively estimated at 83.3% (CI95%: 66.0–87.5%) and 78.1% (CI95%: 62.6–95.3%).

### 3.5. ComBat Harmonization

After harmonization, the AUC of SZE_GLSZM_ in the training cohort remained at 0.82 (*p *< 0.0001) and a new optimized cut-off value of 0.52 was determined, resulting in a BAcc of 77% (Se 72%, Sp 82%) and a significant stratification regarding bRFS (HR: 8.0, *p* < 0.0001).

When applied to the testing cohort after ComBat harmonization, SZE_GLSZM_ values with the same cut-off led to a BAcc of 76% (Se 59%, Sp 93%) and an HR of 5.5 (*p* < 0.0001) regarding bRFS prediction.

Accordingly, in the training cohort, the best “harmonized” clinical + radiomics model was obtained by combining the number of risk factors, postoperative PSA, and the harmonized SZE_GLSZM._ This model had an AUC of 0.82 (*p* < 0.0001), with the optimal cut-off value resulting in a BAcc of 74% (Se 78%, Sp 70%), allowing for significant stratification regarding bRFS (HR: 6.9, *p* < 0.0001). When applied to the testing cohort, this model resulted in a BAcc of 76% (Se 53%, Sp 98%) and an HR of 6.8 (*p* < 0.0001). The “harmonized” models did not significantly differ when compared with the “non-harmonized” models (radiomics only: *p* = 1; radiomics + clinical: *p* = 0.76).

The ROC and corresponding Kaplan–Meier curves corresponding to the “harmonized” prediction models can be found in Figures S2–S4.

The performances of each prediction model according to the cohort are summarized in Table 3.

### 3.6. Inter-Reader Variability

The three independent segmentations were relatively similar, with a mean DICE coefficient of 0.78 and a mean average Hausdorff distance of 0.85 mm (Table S3). The ICC calculated between the ADC SZE_GLSZM_ values corresponding to the different delineations was 0.98 (CI95%: 0.95–0.99) for single measures and 0.99 (CI95%: 0.98–1.0) for average measures. In this subset of patients, no change in BCR prediction occurred with the changes in the ADC SZE_GLSZM_ values across the different delineations (Table S4).

### 3.7. Radiomics Quality Score

Our study scored moderately (17 points out of 36) on the radiomics quality score (Table S5).

## 4. Discussion

To the best of our knowledge, our work is the first to externally validate a radiomics predictive model in the context of high-risk PCa treated by radical prostatectomy only.

Although a couple of clinical features were significantly correlated to BCR, the clinical predictive model combining the association of the number of risk factors and postoperative PSA value failed to provide a significant stratification of patients according to bRFS in the external cohort. The number of risk factors and postoperative PSA are known to be predictive of late BCR with 10 years of follow-up [25,26]. However, this clinical model demonstrated no predictive power in the testing set (balanced accuracy of 56%). By definition, the studied population was already at high-risk of BCR and very homogenous in terms of clinical characteristics; as such, stratification based on clinical features alone among this selected population remains inefficient, thereby emphasizing the need for more robust predictive markers of BCR to tailor postoperative management.

As noted, BCR rates were higher in the external testing cohort of Institution 2 when compared to the training cohort of Institution 1. This could first be explained by the higher percentage of R1 surgical margins among patients with available MRI. Given the low accessibility of MRI in the preoperative setting, one could think that MRIs were reserved for patients at highest risk during presurgical planning. Furthermore, all RPs were performed by one surgeon in Institution 1, whereas several surgeons with a diversity of surgical experiences were involved in Institution 2. Finally, selection bias due to missing MRIs cannot be excluded (Table S1).

A few studies explored radiomics in PCa, including Wibmer et al. who developed an MRI-derived radiomics model for the diagnosis of PCa in 146 patients [27]. Energy, entropy, correlation, and homogeneity were all extracted using the Grey Level Co-occurrence Matrix (GLCM), achieving a significant prediction of the presence of cancer. Similarly, Cameron et al. [28] trained a radiomics signature for PCa detection, which outperformed all other feature sets with an accuracy of 87% (sensitivity 86% and specificity 88%).

Several studies focused on the prediction of clinical outcomes after first-line treatment in PCa. Using the population of two institutions (70 and 50 patients) scanned with two different MRIs, Shiradkar et al. developed a classifier based on radiomics features and clinical variables, reaching an AUC of 0.74 in the testing set [29]. The main limitation of this work was related to the heterogeneous treatment strategies, the absence of patient selection and population comparisons between treatment groups, the small number of studied clinical and histological features, and the lack of data harmonization.

Therefore, we aimed to externally validate our previously developed model relying on a single radiomics feature [14]. We confirmed that this radiomics model was predictive of BCR and could significantly stratify patients according to bRFS based on small zone emphasis (SZE_GLSZM_) calculated using the Grey-Level Small Zone Distance Matrix (GLSZM). The GLSZM analyzes the distance between groups of voxels with similar grey-levels by counting the number of groups of linked voxels, which occur if the neighboring voxel has an identical discretized grey level. SZE focuses on areas of small volume, where the lower the SZE value, the more heterogeneous the intensities in the image (in this case, the ADC map) within the tumor volume are considered to be [18].

The external testing cohort presented several significant differences with the re-training cohort, including MRI parameters, patient characteristics, overall outcome (rate of BCR), and treating physicians. However, despite these differences, the radiomics model performed well, reaching a balanced accuracy of 0.76 and an HR of 5.5. Furthermore, the radiomics model was as effective in the testing cohort as in the retraining cohort, thereby proving its robustness. Its reliability was also supported by the low inter-reader variability of delineation observed in a subset of 15 randomly selected patients, for which no BCR classification changes were induced by the use of a different segmentation.

The testing cohort only included patients scanned using a 1.5T MRI scan, which had lower sensitivity and specificity compared to the 3T MRI scan [30]. Indeed, in the Institution 1 cohort alone, the performance was previously shown to be higher using 3T compared to 1.5T (AUCs of 0.87 and 0.76, respectively), which may contribute to an explanation of why the obtained accuracy was 0.76 [14].

Strict application of international guidelines results in unnecessary treatment in 143/195 (73.3%) of patients. The use of the radiomics model, thanks to a PPV of 83.3% and an NPV of 78.1 (testing cohort), could decrease unnecessary treatment to 32/195 (16.4%) patients overall. The radiomics model was unable to predict BCR in 17/195 patients (8.7%); these patients would be eligible for early sRT at the time of BCR. Although improvement is needed, the model could be useful for better selection of patients eligible for aRT. Such selection is paramount, especially when considering the results of Phase 3 studies comparing early salvage radiotherapy and adjuvant radiotherapy. Preliminary results from RADICALS-RT, GETUG-AFU 17, RAVES, and their meta-analysis (ARTISTIC) [8,9,10] suggested that eSRT was not significantly different from aRT, thereby favoring the use of eSRT instead of aRT.

However, caution is needed regarding the generalizability of these data as aRT may still be beneficial for a subpopulation of patients at high risk of BCR. In fact, rates of Gleason 8–10 PCa in the RADICALS trial were relatively low (17% in the eSRT arm vs. 16% in the aRT arm), as were the rates of seminal vesical invasion (20% in the eSRT arm vs. 19% in the aRT arm). In the pooled analysis of ARTISTIC, similar baseline features were found, with only 9–17% of patients exhibiting Gleason 8–10 PCa and 12–21% showing seminal vesicle invasion. With a 5-year BCR rate of 88% (RAVES trial) compared to 71.4% in our cohort (median follow-up of 41.9 months), the follow-up did not appear sufficient in detecting a benefit of aRT over esRT in a population where the majority only had one high-risk feature. Abdollah et al. showed that the cancer-specific mortality (CSM) rates in patients with zero, one, two, or three high-risk features were 98.6%, 99.6%, 90.3%, and 84.0% at 5 years, respectively [31]. Moreover, even when the PSA is very low (i.e., ≤0.5 ng/mL), pre-radiotherapy PSA is known to be significantly associated with BCR in the eSRT setting (HR: 4.89; CI95%: 1.40–22.9) in populations similar to ours (716 negative-node patients with undetectable postoperative PSA) [32]. Finally, the three trials pooled in the ARTISTIC meta-analysis only focused on BCR-free survival. BCR is known to be a surrogate of clinical outcomes (metastasis-free survival and CSM) [33,34,35]. However a benefit of aRT over esRT on these long-term outcomes might exist, especially given the statistical benefit of aRT on 15-year metastasis-free survival (*p* = 0.036) and the trend toward significance regarding overall survival (*p* = 0.056) [6]. aRT also significantly reduces the need for subsequent androgen deprivation therapy, which is known for its inherent toxicity [36].

Even if these three studies contribute to the better understanding of postoperative PCa relapse, with the subpopulation analysis in the ARTISTIC trial still unpublished, decisions regarding aRT or esRT must remain based on patient characteristics.

Our prediction model, although complex to develop, could be very easily implemented in clinical practice. Indeed, it requires a simple semi-automatic tumor segmentation, which can be rapidly performed by a radiologist using a pre-therapeutic MRI, followed by a fully automated calculation of a single feature (due to our model relying on only one radiomics parameter), allowing for instantaneous predictions for a given patient to be provided to the clinician as soon as the tumor delineation is finished.

With the need for efficient and personalized medicine, our simple model based only on the extraction of a single radiomics feature could comfort clinicians in their decision regarding the best postoperative management with better BCR stratification (PPV of 83.3%).

Interestingly, the ComBat harmonization method did not significantly change our results and the predictions of BCR and bRFS remained similar after harmonizing radiomics features across the scanner models. This method considered all extracted radiomics with the aim to remove the multicenter effect for features heterogeneity of acquisition parameters. While MRI scan parameters differed between the retraining and the external testing cohorts, the radiomics feature performed well, likely reflecting its relatively high robustness with respect to the scanner model, acquisition protocol, and reconstruction setting variability.

The radiomics approach applied to routinely acquired images for diagnosis has the great advantage of being cost-effective and noninvasive. Genomic tests, such as the Decipher Prostate Cancer test^®^ [37], have been used to stratify PCa patients according to metastasis-free survival and cancer-specific mortality. Based on a population of 256 high-risk PCa patients, the c-index of the genomic test was 0.79 (CI95%: 0.68–0.87) [38]. The integration of quantitative imaging data with genomic signatures (radiogenomics) could be of interest in the field of PCa, but very few studies are available to date.

Apart from being a retrospective study, one of the limitations of our study was the absence of PIRADS v2.0 implementation as a potential predictive feature for BCR. PIRADS v2.0 was developed as a diagnostic tool to define and stage malignant tissues (vs. normal prostatic glandular tissue). Even if a correlation with Gleason score is assumed [39], the impact of PIRADS v2.0 as a prediction tool remains unstudied [40]. Furthermore, the short follow-up must be stressed, especially in the context of PCa, as the median follow-up for the nonrelapsing cohort was 50.2 months (range 24–120). However, time from RP to BCR is, on average, 3.5 years [41]. A further analysis with a longer follow-up should be performed to confirm our findings. Some patients in our cohort did not undergo lymphadenectomy. However, the lymph node involvement risk was very low in this subpopulation (median of 5%), with the Roach formula known for overestimating the risk of pelvic nodal invasion [42]. Another potential limitation was the definition of the tumor volumes of interest, which was performed manually by a single expert. Relying on delineations by multiple experts could be performed in future studies to quantify the reproducibility of our findings. Another potential limitation was the definition of the tumor volumes of interest, which was performed manually by a single expert. Nevertheless, when analyzing a subset of 15 patients, our prediction model proved to be reliable regardless of the chosen segmentation, with no BCR classification changes. With a score of 17 on the radiomics quality score, our study scored moderately. The addition of other MRI sequences (such as high b-value DWI sequences and Dynamic Contrast Enhanced sequences) and a prospective validation are currently under investigation at our institution in order to further strengthen the confidence in our findings.

## 5. Conclusions

A radiomics model was externally validated and appeared to be predictive of both BCR and bRFS after RP in patients with high-risk PCa. It seemed robust to patient characteristics and MRI scan variability. This model could help to stratify patients after RP and tailor postoperative management. For patients at high risk of recurrence, intensified postsurgical monitoring or aRT could be offered. on the other hand, patients at very low risk of recurrence could avoid aRT, thus reducing unnecessary treatment and the associated toxicity.

## Figures and Tables

**Table 1:** Patient and tumor characteristics in training and testing cohorts.

Patient Characteristics	Training (%) *n* = 107	Testing (%) *n* = 88	*p*-Value
Age at diagnosis (mean, y)	65.2	66.2	0.25
PSA (mean, ng/mL)	9.3	8.5	0.37
MRI characteristics			
Siemens 1.5T Institution 1 (%)	70.0		
Philips 3T Institution 1 (%)	30.0		
Philips Institution 2 (%)		55.7	
Siemens Institution 2 (%)		44.4	
Surgical characteristics			
Pathological tumor stage			
pT1-pT2	34.6	43.2	0.28
pT3-pT4	65.4	56.8	
Lymph nodes dissection			
yes	68.2	96.6	<0.0001
no	31.8	3.4	
Surgical margins			
R0	40.2	22.7	0.014
R1	58.8	77.3	
Gleason score			
Gleason ≤ 7	86.0	83.0	0.71
Gleason > 7	14.0	17.0	
Median Capra-S Score	4	4	1.00
Mean postoperative PSA (ng/mL)	0.014	0.017	0.22
Median number of risk factors	1	1	
Median bRFS (months)	49.2	33.3	<0.0001
Biochemical recurrence (%)	16.8	38.6	0.0166
Median Follow-up (months)	57.0	41.9	<0.0001

Abbreviations: PSA = prostate specific antigen; MRI = magnetic resonance imaging; bRFS: biochemical relapse-free survival.

**Table 2:** Correlation between clinical/radiomics features and biochemical recurrence (training).

Biochemical Reccurence	Univariate Analysis						Multivariate Analysis	
Feature	AUC	Best Cut-Off	BAcc (%)	Se (%)	Sp (%)	*p*-value	HR	*p*-Value
ADC SZE_GLSZM_	0.82	≤0.53	*79*	72	85	<0.0001	10.9	0.0001
Age at surgery (y)	0.54	>65.7	60	72	48	0.62		
Preoperative PSA (ng/mL)	0.62	>6.5	*64*	78	50	0.08		
Gleason score	0.53	>4	57	17	96	0.72		
T stage	0.62	>3	*58*	78	38	0.07		
Surgical Margins	0.51	>0	*51*	61	41	0.90		
Postoperative PSA (ng/mL)	0.64	>0.01	*63*	56	69	0.04	2.7	0.064
Capra-S Score	0.58	>3	*63*	72	53	0.27		
Number of risk factors	0.64	>1	*64*	56	72	0.04	3.2	0.064

Abbreviations: ADC: Apparent Diffusion Coefficient Map; AUC: Area under the curve; BAcc: Balanced Accuracy; Se: sensitivity; Sp: specificity; HR: Hazard Ratio; SZE: Small Zone Emphasis; GLSZM: Grey-Level Size Zone Matrix.

**Table 3:** Performance of each prediction model according to the cohort.

Prediction Models	BCR Prediction								bRFS Stratification			
	Training					Testing			Training		Testing	
Model	AUC	*p*-Value	BAcc (%)	Se (%)	Sp (%)	BAcc (%)	Se (%)	Sp (%)	HR	*p*-Value	HR	*p*-Value
Clinical	0.68	0.007	63	*78*	47	56	53	*59*	3.2	0.032	1.7	0.19
Radiomics	0.82	<0.0001	78	*72*	84	76	59	*93*	8.7	<0.0001	5.5	<0.0001
C + R	0.86	<0.0001	84	*94*	67	67	91	*43*	25	<0.0001	5.7	<0.0001
Combat R	0.82	<0.0001	77	*72*	82	76	59	*93*	8.0	<0.0001	5.5	<0.0001
Combat C + R	0.82	<0.0001	74	*59*	93	76	53	*98*	6.9	<0.0001	6.8	<0.0001

Abbreviations: BCR: biochemical recurrence; bRFS: biochemical relapse-free survival, C + R: clinical + radiomics; Combat R: harmonized radiomics; Combat C + R: harmonized clinical + radiomics; AUC: area under the curve; BAcc: balanced accuracy; Se: sensitivity; Sp: specificity; HR: hazard ratio.

## Supplementary Materials

The following are available online at https://www.mdpi.com/2072-6694/12/4/814/s1: Figure S1: Receiver operating characteristic (ROC) curves without Combat Harmonization Method in the training cohort (Clinical Model (a), Radiomics Model (b) and Clinical + Radiomics Model (c)); Figure S2: Receiver operating characteristic (ROC) curves after Combat Harmonization Method in the training (a and b) and testing (c and d) cohorts (Radiomics and Clinical + Radiomics Models); Figure S3: Kaplan-Meier estimates of biochemical relapse free survival using the “Combat” radiomics model in the training (a) and testing (b) cohorts; Figure S4: Kaplan-Meier estimates of biochemical relapse free survival using the “Combat” clinical-radiomics model in the training (a) and testing (b) cohorts; Table S1: Summary of MRI scan acquisition parameters; Table S2: Patients and tumors characteristics of the initial population (Institution 1 + Institution 2); Table S3: Inter-reader variability assessment—segmentation; Table S4: Inter-reader variability assessment—BCR-Predicted Probabilities; Table S5: Radiomics Quality Score.
