# Supplementary material for: External Validation of an MRI-Derived Radiomics Model to Predict Biochemical Recurrence after Surgery for High-Risk Prostate Cancer
Source: Cancers (Basel). 2020 Mar 28;12(4):814. doi: 10.3390/cancers12040814 (PMC7226108; doi:10.3390/cancers12040814)

Supplementary material :

Supplementary Table 1: Summary of MRI scan acquisition parameters

Acquisition parameters	Siemens 1.5T (Institution 1)	Philips Achieva 3T (Institution 1)	Siemens 1.5T (Institution 2)	Philips 1.5T (Institution 2)
Number of patients	75	32	37	51
Magnetic field strength (Tesla)	1.5T	3T	1.5T	1.5T
T2-Weighted				
Matrix (pixels)	192 × 192	268 × 268	250 × 250	264 × 264
Field of view (mm)	250 × 250	320 × 320	256 × 256	320 × 320
ET (ms)	110	90	125	110
RT (ms)	2500	4500	2000	3500
Slice Thickness (mm)	1.5	1.5	2	4
ADC map				
Matrix (pixels)	128 × 128	144 × 144	192 × 144	68 × 65
Field of view (mm)	200 × 200	240 × 240	192 × 192	144 × 144
ET (ms)	80	80	95	80
RT (ms)	2300	2300	2500	4000
Slice Thickness (mm)	3.5	3.5	3.5	4
Diffusion gradient	B50-400-1000	B100-600-1000	B50-800	B80-200-400-1100

Abbreviations: RT: repetition time, ET: echo time

Supplementary Table 2: Patients and tumors characteristics of the initial population (Institution 1 + Institution 2)

Institution		Institution 1			Institution 2		
Patients characteristics		Selected patients	Excluded patients because of unavailable MRI	p-value	Selected patients	Excluded patients because of unavailable MRI	p-value
Number of patients		107	30		88	277	
Age at diagnostic (mean.yo)		65.25	*64.5*	*0.56*	66.2	*67.5*	*0.09*
PSA (mean. ng/mL)		9.10	*8.18*	*0.50*	8.47	*8.84*	*0.61*
Post-operative tumour status (%)	pT2	35.5	*46.7*	*0.37*	44.3	*37.5*	*0.31*
	pT3a-pT3b	64.5	*53.3*		55.7	*62.5*	
	pT4	0	*0*		0	*0*	
Nodal status (%)	pN0	87.8	*73.3*	*0.10*	96.6	*96.7*	*0.77*
	cN0	12.2	*26.7*		3.4	*3.3*	
Surgical margins (%)	R0	40.2	*33.3*	*0.64*	22.7	*42.2*	***0.0015***
	R1	58.9	*60*	*0.92*	77.3	*56.7*	***0.0008***
	Rx	0.9	*6.7*	*0.22*	0	*1.1*	*0.75*
Gleason score (%)	Gleason ≤ 7	85.5	*85*	*0.82*	83.0	*82.7*	*0.92*
	Gleason > 7	14.5	*15*		17.0	*17.3*	
Capra-S Score (median)		4	*4*		4	*4*	
Number of risk factors		1	*1*		1	1	
Post-operative PSA (mean. ng/mL)		0.012	*0.017*	*0.07*	0.016	*0.017*	*0.36*
bRFS (median. months)		42.6	*55.3*	*0.06*	33.0	*63.1*	***0.001***
Biochemical recurrence (%)		15.9	*16.1*	*0.49*	38.6	*28.5*	*0.10*
Follow-up (median. months)		52.0	*69.4*	***0.0002***	41.9	*77.7*	***< 0.0001***

Supplementary Table 3: Inter-reader variability assessment - segmentation

PatientID	Hausdorff E1-E2 (mm)	Dice E1 - E2	Hausdorff E1 - E3 (mm)	Dice E1 - E3	Hausdorff E2 - E3 (mm)	Dice E2 - E3
Patient#001	1.17	0.75	0.77	0.8	0.68	0.84
Patient#002	0.58	0.83	0.66	0.8	0.3	0.88
Patient#003	0.45	0.81	0.78	0.77	0.45	0.81
Patient#007	0.74	0.84	0.34	0.92	0.88	0.81
Patient#009	0.84	0.78	1.43	0.68	0.77	0.81
Patient#010	0.97	0.79	0.82	0.81	0.53	0.89
Patient#013	1.24	0.67	1.68	0.56	0.73	0.71
Patient#014	2.70	0.64	2.27	0.66	0.55	0.84
Patient#017	0.97	0.73	0.77	0.76	0.37	0.87
Patient#018	0.74	0.82	1.14	0.76	0.75	0.84
Patient#021	1.34	0.62	0.94	0.70	0.35	0.83
Patient#022	0.59	0.82	0.44	0.81	0.63	0.79
Patient#024	1.31	0.60	0.53	0.69	0.89	0.76
Patient#025	0.82	0.79	0.51	0.84	0.47	0.86
Patient#026	0.95	0.77	0.82	0.80	0.54	0.85

Abbreviations: E1-3: Expert 1-3, ADC : ADC SZE_{GLSZM} value depending on the selected ROI

Supplementary Table 4: Inter-reader variability assessment – BCR predictions

PatientID	Age (y)	PSA preop	Gleason	PSA postop	TR	Margins	Capra	Nb risk Factors	ADC E1	ADC E2	ADC E3	BCR	BCR ADC E1	BCR ADC E2	BCR ADC E3
Patient#001	68	7.35	4 + 3	0.02	T3a	R0	4	1	0.78	0.76	0.77	0	0	0	0
Patient#002	723	14.06	3 + 4	0.01	T3a	R1	6	2	0.70	0.72	0.72	0	0	0	0
Patient#003	65	4.90	4 + 3	0.01	T3b	R1	7	2	0.75	0.74	0.72	1	0	0	0
Patient#007	701	7.65	5 + 5	0.01	T2c	R0	4	1	0.51	0.51	0.51	1	1	1	1
Patient#009	67	7.50	4 + 3	0.03	T3a	R1	6	2	0.65	0.63	0.54	1	0	0	0
Patient#010	61	6.60	3 + 4	0.01	T2c	R1	4	1	0.64	0.67	0.69	0	0	0	0
Patient#013	73	10.00	4 + 4	0.01	T2c	R1	6	2	0.47	0.44	0.43	1	1	1	1
Patient#014	71	6.09	3 + 4	0.01	T3a	R0	3	1	0.76	0.74	0.76	0	0	0	0
Patient#017	54	4.20	3 + 4	0.03	T2c	R1	3	1	0.41	0.39	0.41	1	1	1	1
Patient#018	65	14.00	3 + 3	0.01	T2c	R1	4	1	0.68	0.67	0.70	0	0	0	0
Patient#021	63	7.99	3 + 4	0.01	T3a	R0	3	1	0.43	0.40	0.40	1	1	1	1
Patient#022	71	7.50	4 + 4	0.02	T3a	R0	5	2	0.74	0.71	0.72	0	0	0	0
Patient#024	63	9.40	4 + 3	0.02	T3a	R1	6	2	0.81	0.82	0.81	0	0	0	0
Patient#025	59	7.95	4 + 3	0.02	T3a	R0	4	1	0.77	0.76	0.76	0	0	0	0
Patient#026	62	8.30	5 + 5	0.01	T3b	R1	9	3	0.73	0.73	0.73	0	0	0	0

Abbreviations: BCR: Biochemical Recurrence ground-truth. PSA: Prostate Specific Antigen. Preop: pre-operative. Postop: post-operative. E1-3: Expert 1-3. ADC : ADC SZE_{GLSZM} value depending on the selected delineated volume of interest.

Supplementary Table 5 : Radiomics Quality Score

Item	Points	Current Study
Image protocol quality – well-documented image protocols (e.g.. contrast. slice thickness. energy. etc.) and/or usage of public image protocols allow reproducibility/replicability	+1 (if protocols are well-documented)+1 (if public protocol is used)	2
Multiple segmentations – possible actions are: segmentation by different physicians/algorithms/software. perturbing segmentations by (random) noise. segmentation at different breathing cycles. Analyze feature robustness to segmentation variabilities	+1	1
Phantom study on all scanners – detect inter-scanner differences and vendor-dependent features. Analyze feature robustness to these sources of variability	+1	0
Imaging at multiple time points – collect individuals' images at additional time points. Analyze feature robustness to temporal variabilities (e.g.. organ movement. organ expansion/shrinkage).	+1	0
Feature reduction or adjustment for multiple testing – decreases the risk of overfitting. Overfitting is inevitable if the number of features exceeds the number of samples. Consider feature robustness when selecting features	−3 (if neither measure is implemented)+3 (if either measure is implemented)	3
Multivariable analysis with non radiomic features (e.g.. EGFR mutation) – is expected to provide a more holistic model. Permits correlating/inferencing between radiomics and non radiomics features	+1	1
Detect and discuss biological correlates – demonstration of phenotypic differences (possibly associated with underlying gene–protein expression patterns) deepens understanding of radiomics and biology	+1	0
Cut-off analyses – determine risk groups by either the median. a previously published cut-off or report a continuous risk variable. Reduces the risk of reporting overly optimistic results	+1	1
Discrimination statistics – report discrimination statistics (e.g.. C-statistic. ROC curve. AUC) and their statistical significance (e.g.. p-values. confidence intervals). One can also apply resampling method (e.g.. bootstrapping. cross-validation)	+1 (if a discrimination statistic and its statistical significance are reported)+1 (if also an resampling method technique is applied)	1
Calibration statistics – report calibration statistics (e.g.. Calibration-in-the-large/slope. calibration plots) and their statistical significance (e.g.. p-values. confidence intervals). One can also apply resampling method (e.g.. bootstrapping. cross-validation)	+1 (if a calibration statistic and its statistical significance are reported)+1 (if also an resampling method technique is applied)	1

Prospective study registered in a trial database – provides the highest level of evidence supporting the clinical validity and usefulness of the radiomics biomarker	+7 (for prospective validation of a radiomics signature in an appropriate trial)	0
Comparison to ‘gold standard’ – assess the extent to which the model agrees with/is superior to the current ‘gold standard’ method (e.g.. TNM-staging for survival prediction). This comparison shows the added value of radiomics	−5 (if validation is missing)+2 (if validation is based on a dataset from the same institute)+3 (if validation is based on a dataset from another institute)+4 (if validation is based on two datasets from two distinct institutes)+4 (if the study validates a previously published signature)+5 (if validation is based on three or more datasets from distinct institutes)*Datasets should be of comparable size and should have at least 10 events per model feature.	4
Potential clinical utility – report on the current and potential application of the model in a clinical setting (e.g.. decision curve analysis)	+2	2
Cost-effectiveness analysis – report on the cost-effectiveness of the clinical application (e.g.. quality adjusted life years generated)	+2	2
Open science and data – make code and data publicly available. Open science facilitates knowledge transfer and reproducibility of the study	+1	0
Comparison to ‘gold standard’ – assess the extent to which the model agrees with/is superior to the current ‘gold standard’ method (e.g.. TNM-staging for survival prediction). This comparison shows the added value of radiomics	+1 (if scans are open source)+1 (if region of interest segmentations are open source)+1 (if code is open source)+1 (if radiomics features are calculated on a set of representative ROIs and the calculated features + representative ROIs are open source)	0
Total	36	18

Supplementary Figure 1: Receiver operating characteristic (ROC) curves without Combat Harmonization Method

A: clinical model - training

B: radiomic model - training

C: clinical – radiomic model - training

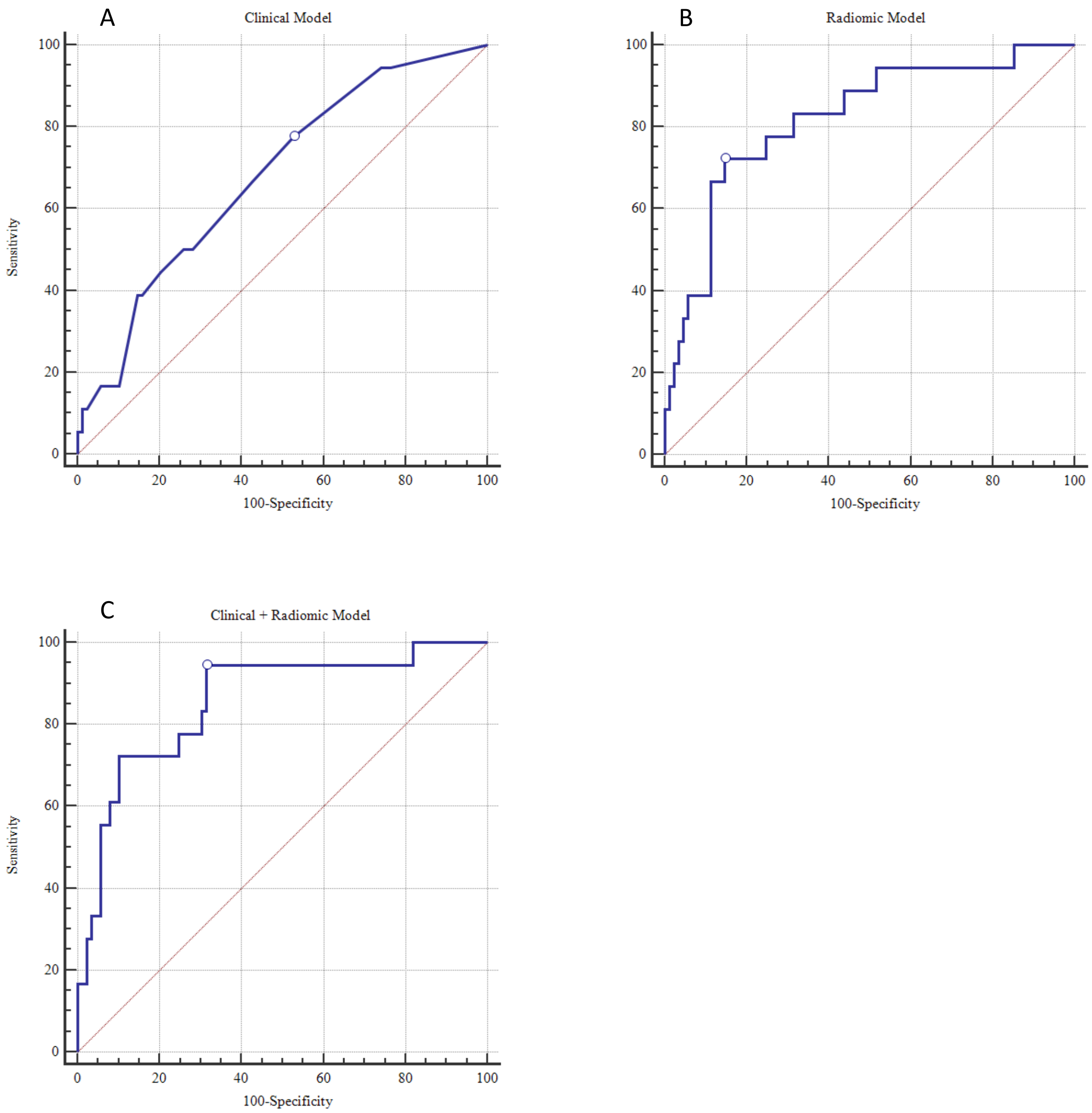

Supplementary Figure 2: Receiver operating characteristic (ROC) curves after Combat Harmonization Method

A: radiomic model – training

B: clinical – radiomic model – training

C: radiomic model – testing

D: clinical – radiomic model - testing

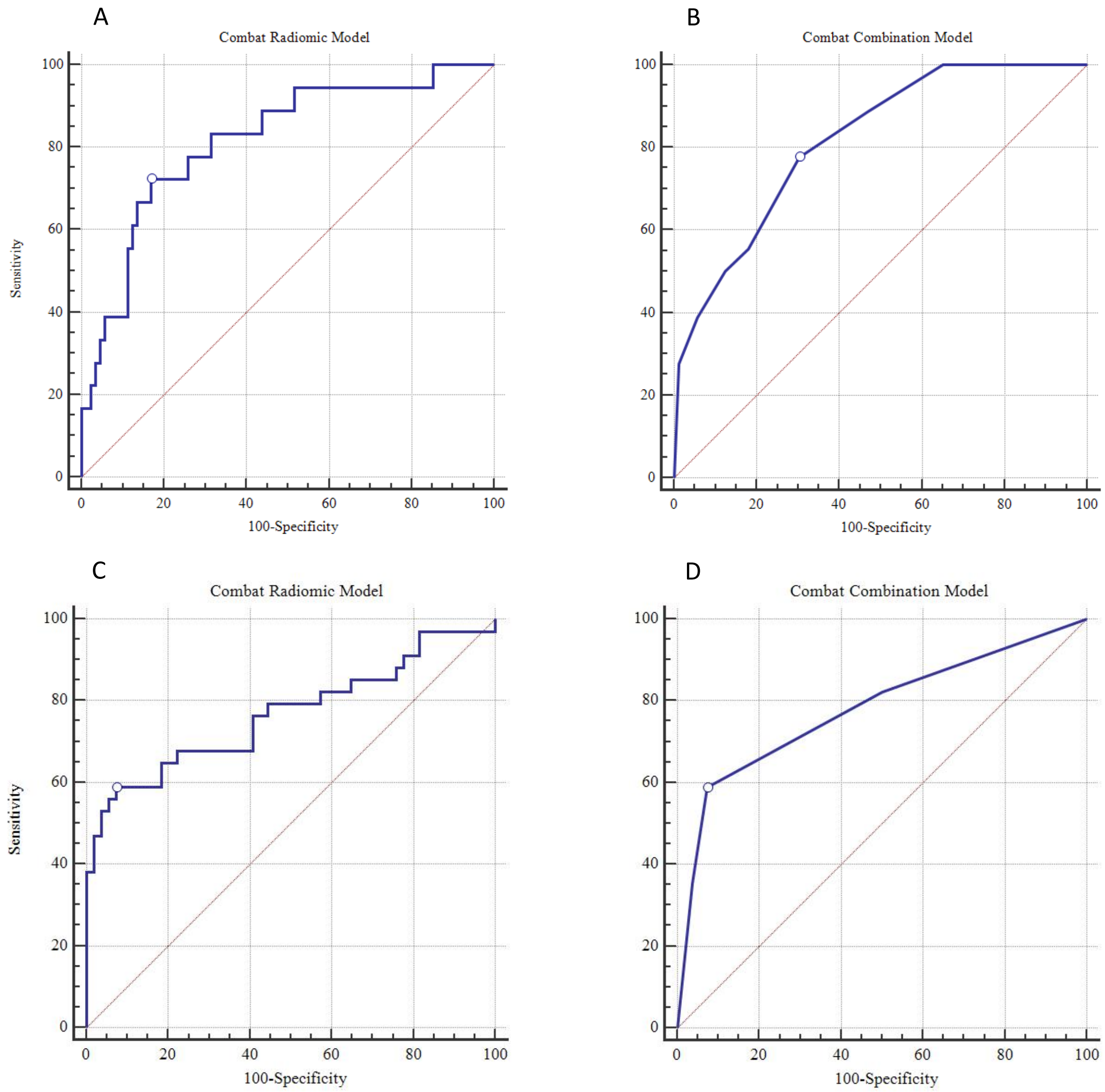

Supplementary Figure 3: Kaplan-Meier estimates of biochemical relapse free survival using the "Combat" radiomic model in the training (a) and testing (b) cohorts.

Figure 3a

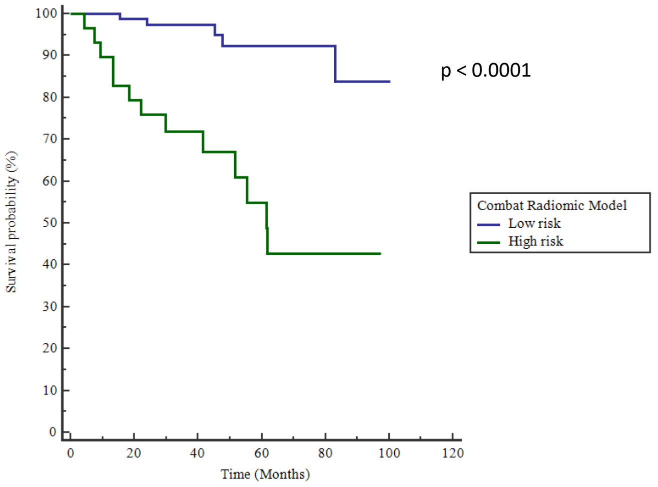

Figure 3b

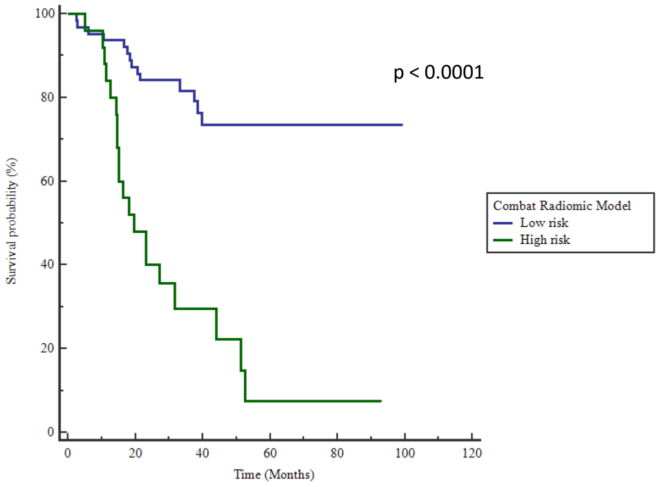

Supplementary Figure 4: Kaplan-Meier estimates of biochemical relapse free survival using the "Combat" clinical-radiomic model in the training (a) and testing (b) cohorts.

Figure 4a

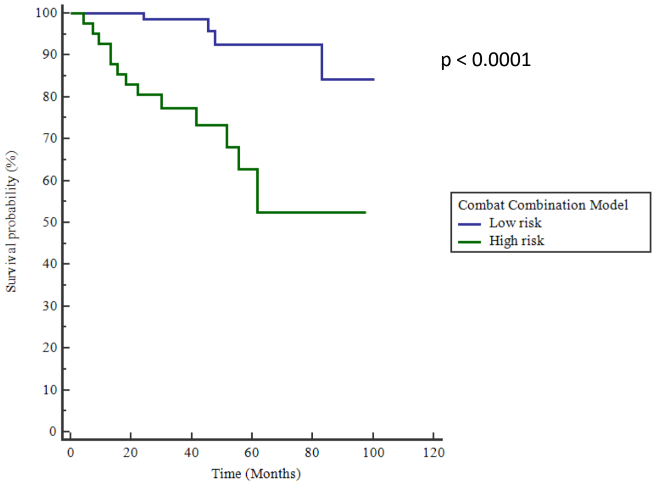

Figure 4b

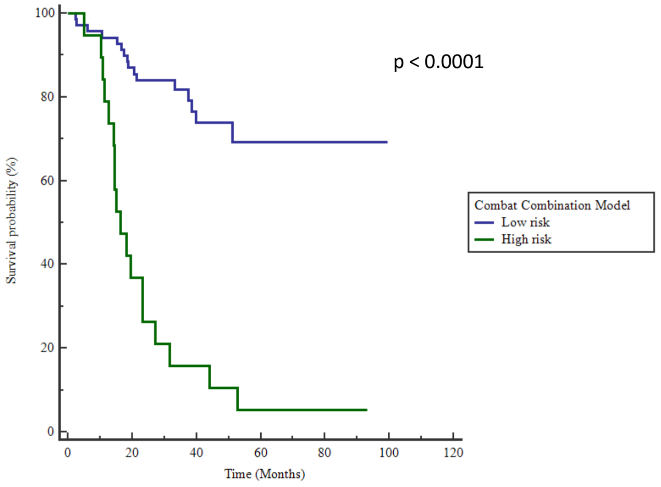

Supplement: Supplementary file 1 [file cancers-12-00814-s001.pdf]
